# Switchable Multicolor Single-Mode Lasing in Polymer-Coupled Microfibers

**DOI:** 10.3390/polym17212917

**Published:** 2025-10-31

**Authors:** Kun Ge, Zishu Zhou, Songtao Li

**Affiliations:** 1 College of Optical and Electronic Technology, China Jiliang University, Hangzhou 310018, China; kunge@cjlu.edu.cn; 2 Department of Mathematics & Physics, North China Electric Power University, Baoding 071003, China; 3 College of Mathematics and Physics, North China Electric Power University, Beijing 102206, China

**Keywords:** multicolor, single mode lasing, polymer, microfibers

## Abstract

Switchable microlasers with multicolor output and high spectral purity are of crucial importance for various photonic devices. However, switchable multicolor lasing usually operates in multimode, which largely restricts its practical applications due to the lack of an effective mode selection mechanism. Here, switchable single-mode lasing was successfully achieved in coupled microfiber cavities, in which each microfiber served as both WGM resonator and mode filter for another microfiber. The unique mode selection mechanism is demonstrated experimentally and theoretically in the coupled microfibers. Furthermore, the color of single-mode lasing is tunable at will via the doping of microfibers with different active materials. Our work might provide a platform for building switchable multicolor lasers and gaining further insights into photonic integration.

## 1. Introduction

Switchable multicolor whispering gallery mode (WGM) lasing is an important potential application in sensing, display and optoelectronics, with profound implications for next-generation sensing, high-resolution display, and visible light communication technologies [VLC] [1,2,3]. In sensing applications, the ability to switch from different lasing wavelengths enables the simultaneous detection of multiple analytes with high specificity; each target molecule triggers a distinct wavelength shift that can be precisely identified. In display technologies, multicolor WGM lasing holds promise for ultra-high-pixel-density devices, as the compact cavity size of WGM resonators allows for miniaturized pixel arrays beyond the limitations of traditional light-emitting diodes. For light communication technology systems, coverage of the entire visible wavelength band is essential for achieving high data transmission rates, making switchable multicolor lasing a key component for achieving broadband optical communication [4].

Most wavelength-switchable microlasers are subject to multicolor light due to the lack of a mode selection mechanism, thus leading to false signaling [4,5]. To mitigate the issue of false signals and enable reliable operation, extensive efforts have been devoted to developing single-mode lasing strategies, thus leveraging diverse physical mechanisms. Distributed feedback (DFB) gratings, for instance, utilize periodic refractive index modulations to form a feedback loop that selectively amplifies a single resonance mode, achieving narrow-linewidth single-mode emission [6,7,8]. However, DFB-based devices typically require precise nanofabrication of grating structures, increasing manufacturing complexity and costs [8]. Self-coupled resonators exploit the interference between coupled cavity modes to suppress unwanted modes, but their performance is highly sensitive to cavity alignment and environmental perturbations [9,10,11]. The parity-time (PT) symmetry breaking mechanism, which relies on balanced gain and loss distributions, enables single-mode lasing by lifting mode degeneracy, yet it demands strict control over the gain–loss balance, which is challenging to maintain in dynamic operating conditions [12,13,14]. The Vernier effect, achieved by coupling two resonators with slightly different free spectral ranges, amplifies the mode spacing of the combined system to isolate a single lasing mode [15,16,17,18,19,20]; while effective, this approach often introduces additional cavity components, increasing the overall device footprint.

Generally, microcavities have proven advantageous for single-mode lasing, as their small mode volume restricts the number of supported resonant modes and enhances mode competition. Materials such as silica microspheres, perovskite microdisks, and semiconductor nanowires have been widely adopted as micro–nano cavities due to their excellent optical confinement and low propagation loss [21,22]. However, most of the strategies allow the achievement of single-mode lasing in one gain region, which restricts WGM lasing [23,24,25,26] of the application in multicolor lasing displays and full visible wavelength band communication. Currently, red–green–blue (RGB) microlasers are achieved by integrating different gain media in the microcavity [27,28]. However, increasing the size of microcavities is not conducive to the development of integration optoelectronic devices [29]. Moreover, the uneven distribution of different gain media within the cavity can lead to unequal lasing thresholds for RGB modes, further complicating the realization of stable, switchable single-mode emissions across the full visible spectrum.

Herein, we demonstrate an effective mode selection mechanism to achieve switchable multicolor single-mode lasing in polymer-coupled microfibers. The coupling microstructure consists of two polymer microfibers, and the polymer microfibers are fabricated by the pultrusion spinning technique. The Q factor of the simple microfiber is over 10^4^. The results prove that the microfibers can be used for high-sensitivity detection. In the microfiber-coupled system, each isolated microfiber serves as not only the WGM resonator but also the mode filter for another microfiber. Microcavities with perfect boundaries and smooth surfaces could be applied as WGM resonators. Moreover, switchable single-mode lasing can be successfully achieved in the coupled microfiber cavities, with each microfiber serving as both a WGM resonator and mode filter for another microfiber.

## 2. Methodology

**Figure 1:**
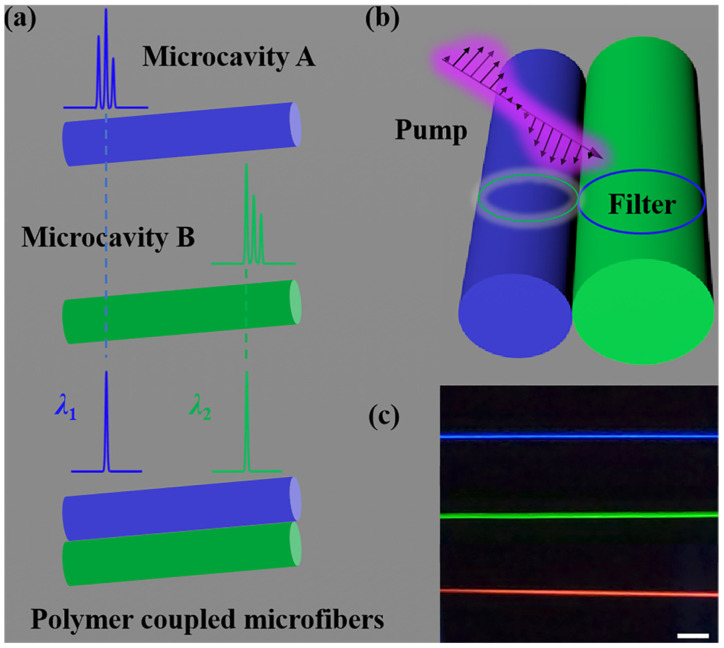
Mode selection concept for the coupled microfibers. (**a**) The multimode lasing from isolated microfibers A and B (top and middle) and switchable single-mode lasing from coupled fiber microcavities (bottom). (**b**) The microfibers serve as both the WGM lasing site and the mode filter for each other. (**c**) The PL images of the microfibers. Scale bar is at 100 μm.

The mode selection concept for switchable multicolor single-mode lasing is shown in Figure 1a. All the dye-doped polymer microfibers have smooth surfaces, and can act as excellent resonant microcavities. The RGB whispering gallery mode lasers were obtained by doping the corresponding active materials. As shown at the bottom of Figure 1a, each microfiber can simultaneously serve as a laser source and a modulator, allowing single-mode lasing to be achieved in the polymer coupled microfibers. For instance, when the coupling microfiber is optimized to resonate at the green WGM lasing wavelength, it will efficiently transmit green light while filtering out red and blue components, resulting in stable green single-mode lasing. Conversely, reconfiguring the coupling microfiber’s parameters to match the red or blue resonant wavelengths involves switching the lasing output to the corresponding color. This switching is rapid and reversible, as the micromanipulator allows for dynamic adjustment of the coupling geometry without altering the intrinsic properties of the emissive WGM lasers. Moreover, switchable multicolor single-mode lasing may be achieved by altering typical coupling microfibers. The single-mode operation is preserved across all color states due to the combined effects of the emissive microfiber’s intrinsic mode suppression and the coupling cavity’s narrow transmission bandwidth. This makes the system highly attractive for applications such as high-resolution displays, and optical communication and sensing. Here, the RGB-emissive microfibers were coupled with the micromanipulator. The dye-doped microfibers can enable WGM lasing, while the coupled microfiber cavities act as filters, as shown in Figure 1b.

Materials. To fabricate the polymer microfibers, three typical light-emitting active materials of Disodium 4, 4′-Bis (2-sulfonatostyryl)biphenyl (S420), Fluorescein sodium salt (uranin), and Rhodamine B (RhB) were adopted as the dye material in the experiment. The chemical reagents serve as matrix, which included three light-emitting molecules RhB (Tianjin Fuchen Chemical Reagents Factory, Tianjin, China), uranin (A46092, Shaen (Shanghai, China) Chemical Technology Co., Ltd., Shanghai, China), S420 (D-36543, Tianjin Heowns Biochem LLC, Tianjin, China), and Polyvinyl alcohol (PVA, S27039-500g, Beijing Hong Hu United Chemical Products Co., Ltd., Beijing, China).

Fabrication. The PVA solution is formed by dissolving PVA in deionized water with a concentration of 16 wt%. The S420, uranin, and RhB are dissolved in deionized water at concentrations of 10 mg/mL and 9 mg/mL, 6 mg/mL, respectively. The PVA solution is formed by dissolving PVA in deionized water at a concentration of 16 wt%. The solution is completely dissolved under heating and magnetic stirring. The polymer microfibers are obtained by the pulling method.

Micromanipulation. Micromanipulation is carried out by using a taper probe (Jiayong Technology Co., Ltd., Shenzhen, China) mounted on a precisely controlled three-dimensional moving stage under an optical microscope (Daheng Optics, Beijing, China). The multicolor-coupled microfibers can be controlled by using the taper probe. To identify the polymer microfiber, the microfiber is excited with a µ-PL operating system (FST2-MPL-400L1, Zhuoli Han Guang Instrument Co., Ltd., Beijing, China) under a nanosecond laser at a wavelength of 343 nm (third harmonics from a 1030 nm Yb:YAG laser, repetition frequency of 200 Hz, and pulse width of 1 ns). The laser beam is adjusted to fully cover the whole sample. The microfibers doped with RhB, uranin, and S420 emitted uniform red, green, and blue fluorescence, respectively, under ultraviolet (UV) excitation as displayed in Figure 1c. The dye materials emit photoluminescence (PL) at blue, green, and red wavebands (Supplementary Materials).

## 3. Results

**Figure 2:**
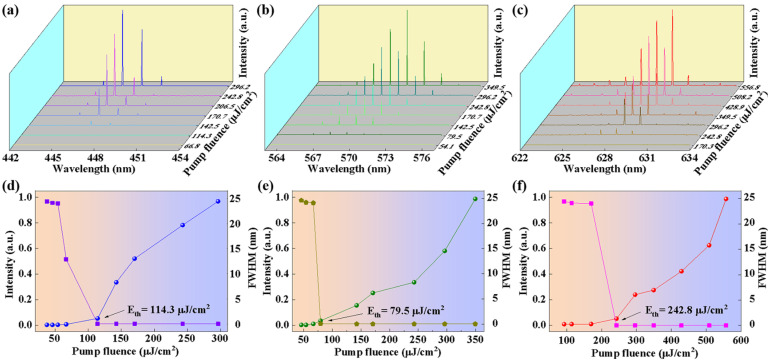
Modulation of the emission spectral. Pumping-power-dependent lasing spectra of the microfibers that are (**a**) blue-emissive, (**b**) green-emissive, and (**c**) red-emissive, respectively. The relationship between emission peak intensity and FWHM under different pump fluence values. Threshold behavior of polymer microfibers at (**d**) 114.3 μJ/cm^2^, (**e**) 79.5 μJ/cm^2^, and (**f**) 242.8 μJ/cm^2^, respectively.

A pulsed laser beam (343 nm, 200 Hz, 1 ns) was used in the experiment. When microfiber was pumped, the response of the WGM laser was recorded. As shown in Figure 2a–c, the WGM lasing emission spectra was realized. Figure 2a–c shows the lasing profiles of three dye-doped microfibers that are blue-emissive (Figure 2a), green-emissive (Figure 2b), and red-emissive (Figure 2c), respectively. Furthermore, the relationship between emission peak intensity and full width at half maximum (FWHM) under different pump fluence values are shown in Figure 2d–f. The threshold behavior of polymer microfibers at 114.3 μJ/cm^2^, 79.5 μJ/cm^2^ and 242.8 μJ/cm^2^ are demonstrated for those that are blue-emissive (Figure 2d), green-emissive (Figure 2e), and red-emissive (Figure 2f), respectively. The results indicate that the microfiber cavities have smooth surfaces and lower optical losses (Supplementary Materials).

**Figure 3:**
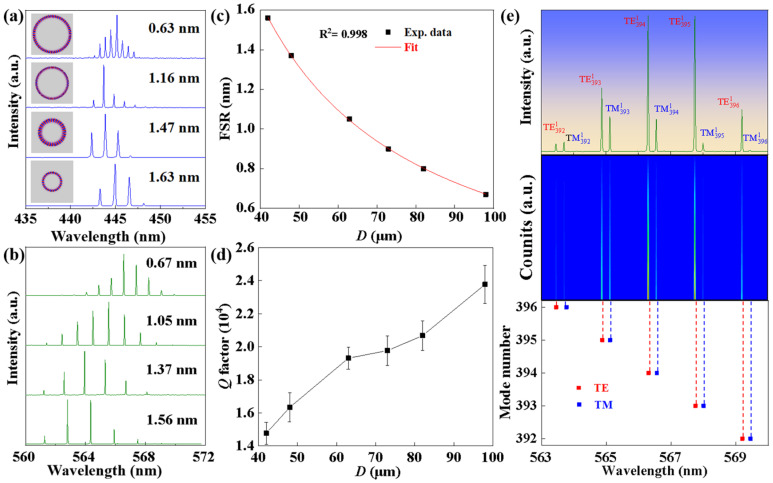
Size-dependent lasing spectra of the polymer microfibers. The WGM lasing modulation with different diameters for (**a**) blue-emissive microfibers and (**b**) green-emissive microfibers. Bottom left illustration shows the simulated electric field intensity in transverse cross-section. (**c**) The relationship between FSR and diameter. (**d**) The Q factor with different diameters. (**e**) The lasing spectra is from Equation (2).

The free spectrum range (FSR) values for blue-emissive microfibers are 0.63 nm, 1.16 nm, 1.47 nm, and 1.63 nm, and the values for the green-emissive microfibers are 0.67 nm, 1.05 nm, 1.37 nm, and 1.56 nm, as displayed in Figure 3a and Figure 3b, respectively. Additionally, the line-widths (Δ*λ*) are less than 0.01 nm. To obtain more information about the WGM lasing between the FSR and the sizes of the microcavities, six sets of lasing spectra with different diameters were recorded, as shown in Figure 3c. Hence, according to the WGM theory [30,31,32]:

(1) $$FSR=\frac{\lambda^{2}}{\pi Dn_{eff}}$$

Here, *λ* is the peak wavelength, *D* represents microfiber diameter, and *n*_eff_ is the effective refractive index, respectively.

As shown in Figure 3c, there is an inversely proportional relationship between the FSR and diameter. The results demonstrated that the combination of experimental methods and theory is very well-matched.

The quality (*Q*) factor can be tuned by changing the size of microfibers. The *Q* factor can be estimated by using the equation *Q* = λ/Δλ, where λ is the center wavelength and Δλ is the line-widths of the lasing mode [33,34]. As displayed in Figure 3d, the *Q* factor of polymer fiber microcavities is over 10,000 (Supplementary Materials), which indicates that the microfiber cavity has suffered minor optical loss. It might be applicable in situations requiring high-sensitivity sensing and detection (Supplementary Materials). According to the WGM equation [35,36,37,38,39]:

(2) $$m\lambda_{m}=\pi Dn_{eff}$$

where *λ*_m_ is the peak wavelength and m is the mode number, respectively.

## 4. Discussion

**Figure 4:**
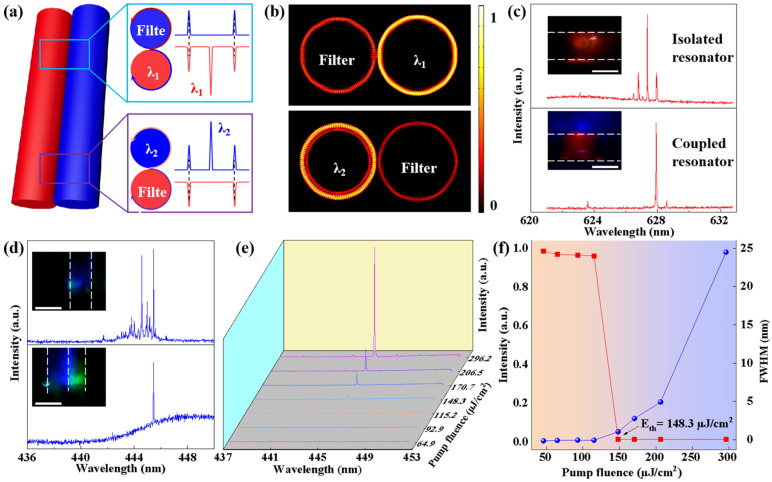
Switchable multicolor single-mode lasing. (**a**) Schematic illustration for achieving single-mode lasing. (**b**) Numerically simulated electric field distributions. Lasing spectra of (**c**) RhB-doped microfiber and (**d**) S20-doped microfibers. Insets: Corresponding PL images of the isolated microfiber and coupled microfibers. Scale bars are at 50 μm. (**e**) The lasing spectra with different pump fluence values for the coupled microfibers. (**f**) The threshold behavior of single-mode lasing is 148.3 μJ/cm^2^.

To achieve single-mode lasing, the mode selection mechanism is used in the coupled microfibers, as shown in Figure 4a. The mode selection mechanism of coupled microcavities is to make the optical field of a specific frequency meet the resonance condition and be amplified through intercavity coupling, while suppressing other frequency modes. Only the modes that satisfy the phase-matching and frequency resonance conditions can stably exist in the coupled microcavity. In the experiment, when the blue-emissive microfiber serves as the lasing cavity, single-mode lasing at the green waveband can be realized with the coupling of a filter cavity. The mode selection mechanism is used to achieve single-mode lasing emission at different wavebands, which is supported by the simulated electric field distributions. This result indicates that the mode selection mechanism could be used to generate multicolor single-mode lasing.

The coupled structure consists of an active gain microcavity and a passive microcavity, whose evanescent fields overlap to form a mutual coupling effect. The passive microcavity, with its well-defined resonant frequency response, can serve as a narrow-band filter of the lasing modes in the active microcavity [38,39]. It introduces significant radiation loss for most non-resonant modes while providing low-loss transmission for the mode that matches its resonant frequency. Thus, single-mode lasing will be achieved at this resonant cavity (Figure 4a, top), due to the loss introduced by the filter cavity. When the blue-emissive microfiber serves as the lasing cavity, single-mode lasing in the green waveband can be realized with the coupling of a filter cavity (Figure 4a, bottom). The mode selection mechanism is used to achieve single-mode lasing emissions at different wavebands (Supplementary Materials), which is supported by the simulated electric field distributions as displayed in Figure 4b. This result indicates that the mode selection mechanism could be used to generate multicolor single-mode lasing.

Micro/nano-manipulation technology focuses on the precise positioning, assembly, and performance control of micro/nano-scale objects. It is the key to realizing orderly construction and functional integration of micro/nano structures. This manipulation technology mainly relies on advanced equipment such as optical microscopes and high-precision three-dimensional translation stages. It enables the grasping, stretching, and bending deformation of individual nano- or microfibers. The micromanipulation is carried out by using a taper probe mounted on a precisely controlled three-dimensional moving stage under an optical microscope. The multicolor-coupled microfibers can be controlled by using the taper probe. The isolated RhB-doped microfiber exhibited activity in the multimode lasing spectrum (Figure 4c, top). When one of the lasing modes was selectively applied in the coupled resonator, single-mode lasing was achieved (Figure 4c, bottom). Such a phenomenon could also be found in the blue-emissive microfibers, as shown in Figure 4d. The spectral characteristics of single-mode lasing were explored. Figure 4e revealed the relationship between lasing spectra and pump fluence in the coupled microfibers, for which the threshold behavior of single-mode lasing is 148.3 μJ/cm^2^, as shown in Figure 4f.

There has been a series of technological breakthroughs in the study of microfibers, particularly in the development of multicolor single-mode lasers, which has significantly contributed to state-of-the-art advancements in optical communication, laser displays, and wearable devices.

Optical communication. Multicolor lasers can transmit multiple optical signals of different wavelengths simultaneously, greatly increasing the capacity and spectral efficiency. Multicolor single-mode lasers based on the microfiber mode selection mechanism can accurately generate multicolor single-mode laser output, reducing crosstalk between signals of different wavelengths and ensuring communication quality. They provide key light source support for high-speed, large-capacity optical communication networks [40,41,42].

Laser display. Full-color single-mode lasers can provide high-purity single-mode lasers of primary colors. Multicolor single-mode lasers fabricated based on the microfiber mode selection mechanism offer a new approach to achieving high-efficiency full-color laser displays. They can enhance the vividness of color, contrast, and resolution of display devices, boasting broad application prospects [43,44].

Wearable devices. The microfiber mode selection mechanism contributes to the miniaturization and integration of multicolor single-mode lasers, allowing them to be integrated with other photonic devices on the same chip to build multifunctional integrated photonic chips. This is of great significance for realizing on-chip optical signal processing, optical computing, and other functions. It can drive the development of integrated photonics technology and meet the demand for miniaturized, high-performance optoelectronic devices in future information technology systems [45,46,47].

## 5. Conclusions

In summary, we have developed a general strategy for achieving switchable multicolor lasing based on a single-mode selection mechanism for polymer-coupled microfibers. This method provides an effective and simple strategy for realizing multicolor single-mode lasing. Specifically, we designed a whispering gallery mode microcavity via a facile self-assembly approach; this coupled microcavity consists of multicolor polymer microfibers. Additionally, the *Q* factor of the simple microfiber is over 10^4^. The results provide evidence that microfibers can be used for systems requiring high-sensitivity detection. Multicolor single-mode lasing was ultimately achieved based on a mode selection mechanism. The full-color single-mode laser can be successfully achieved by coupling multicolor microfibers. Moreover, microfiber microcavities can be combined with weaving technology, which provides a reference for the next generation of flexible wearable devices.

## Data Availability

The original contributions presented in this study are included in the article/supplementary material. Further inquiries can be directed to the corresponding author.

## Supplementary Materials

The following supporting information can be downloaded at https://www.mdpi.com/article/10.3390/polym17212917/s1, Figure S1: The absorption and PL emission spectra of three active materials with S420, uranin and RhB; Figure S2: The multimode lasing spectra from microfibers doped with S420, uranin and RhB, excited with a pulsed laser; Figure S3: The *Q* factor for blue-emissive microfiber, green-emissive microfiber and red-emissive microfiber; Figure S4: The multimode lasing spectra with TE mode and TM mode; Figure S5: The WGM lasing spectra with isolated microfiber and single mode lasing in coupled microfibers.
