# Supplementary material for: Switchable Multicolor Single-Mode Lasing in Polymer-Coupled Microfibers
Source: Polymers (Basel). 2025 Oct 31;17(21):2917. doi: 10.3390/polym17212917 (PMC12608600; doi:10.3390/polym17212917)
Supplement: Supplementary file 1 [file polymers-17-02917-s001.zip › polymers-3846714-supplementary.pdf]

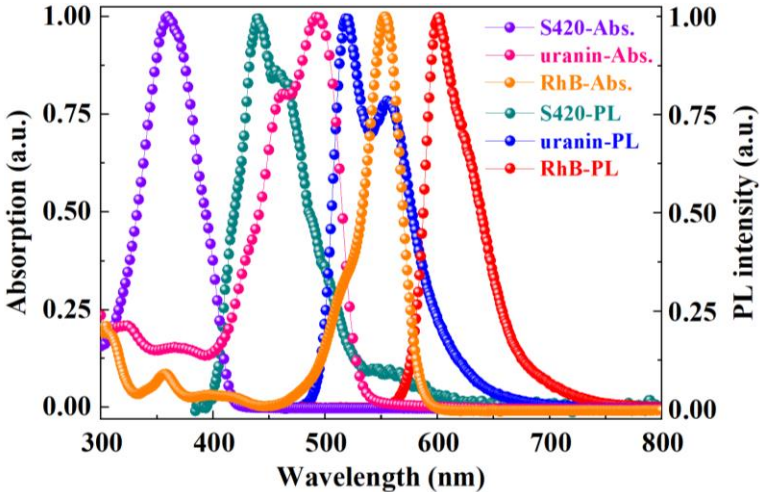

Figure S1. The absorption and PL emission spectra of three active materials with S420, uranin and RhB. Figure S1 presents the absorption and PL emission spectra of the three active materials in deionized water span the RGB color. And the maximum emission peak wavelength are at ~440 nm, ~550 nm and ~600 nm, respectively. Thus, we can achieve the microlasers in visible light wavebands.

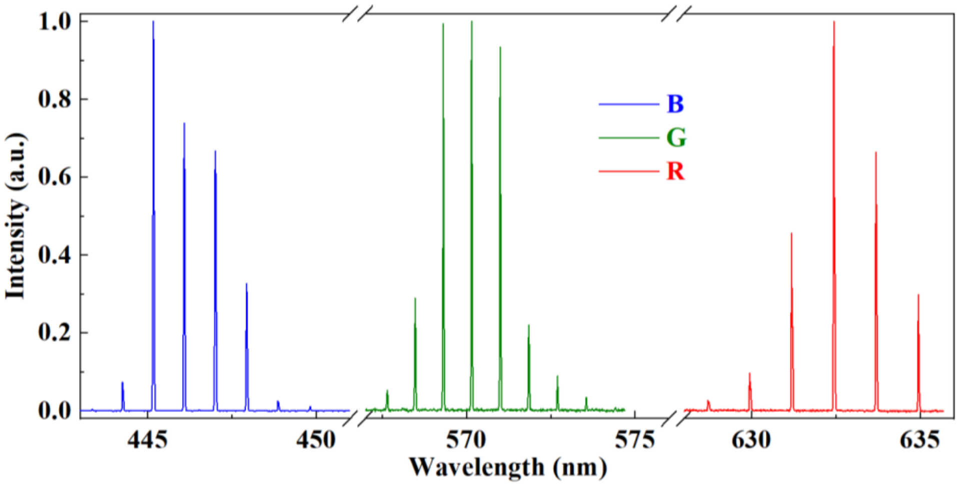

Figure S2. The multimode lasing spectra from microfibers doped with S420, uranin and RhB, excited with a pulsed laser (343 nm). Switchable microlasers are cover whole visible spectrum. As shown in Figure S2, the multicolor WGM lasing can be obtained when the dye-doped isolated polymer microfibers were excited with a pulsed laser.

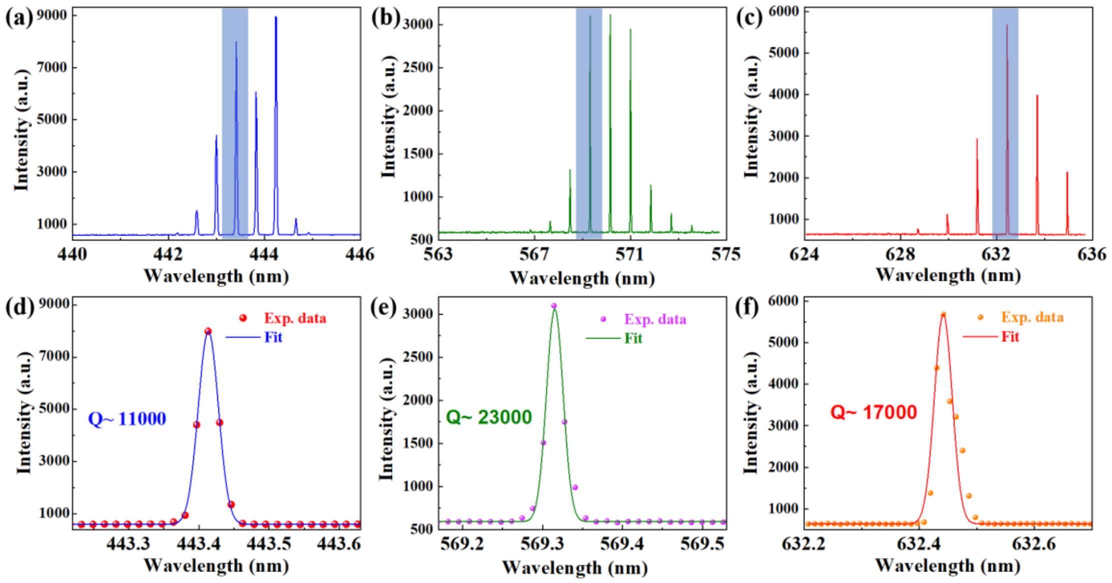

Figure S3. The Q factor for blue-emissive microfiber, green-emissive microfiber and red-emissive microfiber. Figure S3 demonstrates the multimode output in micorfiber cavities with different wavebands. The quality (Q) factor is a fundamental physical parameter in WGM microcavity, which is used to describe the speed of energy attenuation or its ability to store energy. The Q factor can be written as:

$$Q = \omega \frac{U}{P} = \omega \frac{U}{-dU/dt} \tag{S1}$$

here, U represents the total energy stored inside the microcavity; P=−dUdt is the energy loss per unit time, i.e. the power loss; ω is the resonant circular frequency.

For WGM resonant microcavity, the line-width is one of the parameters for detection limit of WGM sensor. The expression for the Q factor is:

$$Q = \omega\tau = \frac{\lambda}{\Delta\lambda} \tag{S2}$$

here, τ is the decay time of the stored energ; λ represents the resonant wavelength; $\Delta\lambda$ is the full width half maximum. And the Q factors are over 10000, which $\Delta\lambda$ is less than 0.1 nm. The results provide that the microfibers can be used to high sensitivity detection

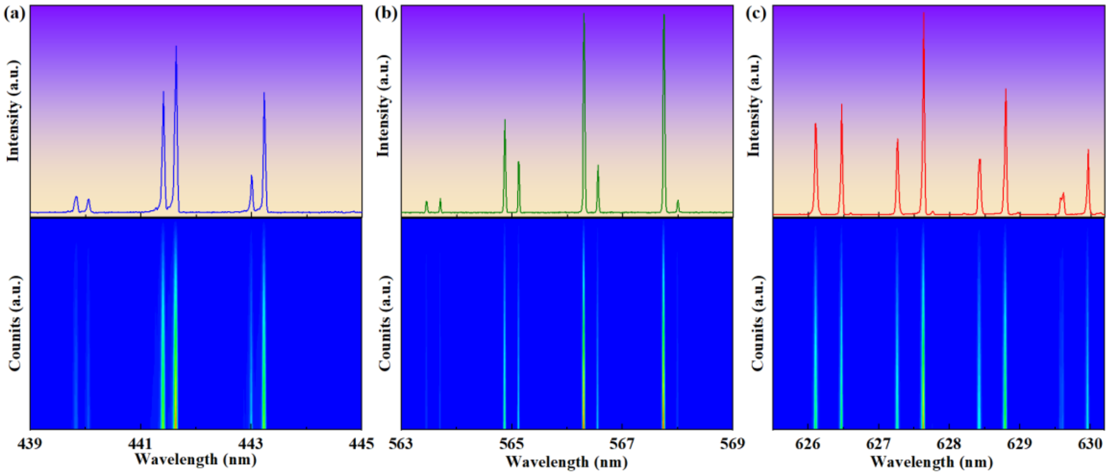

Figure S4. The multimode lasing spectra with TE mode and TM mode.

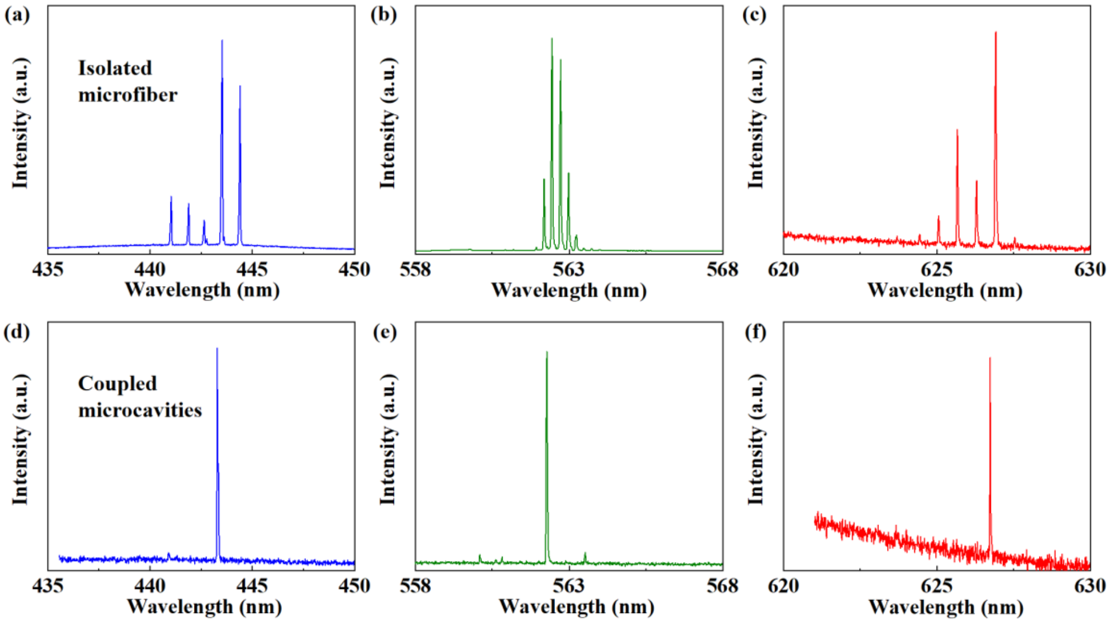

Figure S5. The WGM lasing spectra with isolated microfiber and single mode lasing in coupled microfibers. The multimode lasing can be achieved in isolated microfibers as shown in Figure S5. The switchable single mode lasing can be successfully achieved in the coupled microfibers cavities, which each microfiber were server as both WGM resonator and mode filter for another microfiber.
